# Association of Socioeconomic Status With Dementia Diagnosis Among Older Adults in Denmark

**DOI:** 10.1001/jamanetworkopen.2021.10432

**Published:** 2021-05-18

**Authors:** Jindong Ding Petersen, Sonja Wehberg, Aake Packness, Nanna Herning Svensson, Nana Hyldig, Søren Raunsgaard, Merethe Kirstine Andersen, Jesper Ryg, Stewart W. Mercer, Jens Søndergaard, Frans Boch Waldorff

**Affiliations:** 1 Research Unit of General Practice, Department of Public Health, University of Southern Denmark, Odense, Denmark; 2 Psychiatric Research Unit, Psychiatry Region Zealand, Slagelse, Denmark; 3 OPEN (Open Patient Data Explorative Network), Odense University Hospital, Region of Southern Denmark, Odense, Denmark; 4 Geriatric Research Unit, Department of Geriatric Medicine, Odense University Hospital, Odense, Denmark; 5 Department of Clinical Research, University of Southern Denmark, Odense, Denmark; 6 Advanced Care Research Centre, Usher Institute, University of Edinburgh, Edinburgh, Scotland; 7 Section of General Practice, Research Unit of General Practice, Department of Public Health, University of Copenhagen, Copenhagen, Denmark

## Abstract

**Importance:**

Low socioeconomic status (SES) has been identified as a risk factor for the development of dementia. However, few studies have focused on the association between SES and dementia diagnostic evaluation on a population level.

**Objective:**

To investigate whether household income (HHI) is associated with dementia diagnosis and cognitive severity at the time of diagnosis.

**Design, Setting, and Participants:**

This population- and register-based cross-sectional study analyzed health, social, and economic data obtained from various Danish national registers. The study population comprised individuals who received a first-time referral for a diagnostic evaluation for dementia to the secondary health care sector of Denmark between January 1, 2017, and December 17, 2018. Dementia-related health data were retrieved from the Danish Quality Database for Dementia. Data analysis was conducted from October 2019 to December 2020.

**Exposures:**

Annual HHI (used as a proxy for SES) for 2015 and 2016 was obtained from Statistics Denmark and categorized into upper, middle, and lower tertiles within 5-year interval age groups.

**Main Outcomes and Measures:**

Dementia diagnoses (Alzheimer disease, vascular dementia, mixed dementia, dementia with Lewy bodies, Parkinson disease dementia, or other) and cognitive stages at diagnosis (cognitively intact; mild cognitive impairment but not dementia; or mild, moderate, or severe dementia) were retrieved from the database. Univariable and multivariable logistic and linear regressions adjusted for age group, sex, region of residence, household type, period (2017 and 2018), medication type, and medical conditions were analyzed for a possible association between HHI and receipt of dementia diagnosis.

**Results:**

Among the 10 191 individuals (mean [SD] age, 75 [10] years; 5476 women [53.7%]) included in the study, 8844 (86.8%) were diagnosed with dementia. Individuals with HHI in the upper tertile compared with those with lower-tertile HHI were less likely to receive a dementia diagnosis after referral (odds ratio, 0.65; 95% CI, 0.55-0.78) and, if diagnosed with dementia, had less severe cognitive stage (β, −0.16; 95% CI, −0.21 to −0.10). Individuals with middle-tertile HHI did not significantly differ from those with lower-tertile HHI in terms of dementia diagnosis (odds ratio, 0.92; 95% CI, 0.77-1.09) and cognitive stage at diagnosis (β, 0.01; 95% CI, −0.04 to 0.06).

**Conclusions and Relevance:**

The results of this study revealed a social inequality in dementia diagnostic evaluation: in Denmark, people with higher income seem to receive an earlier diagnosis. Public health strategies should target people with lower SES for earlier dementia detection and intervention.

## Key Points

**Question:**

Is socioeconomic status, as defined by household income, associated with dementia diagnosis and cognitive severity at diagnosis?

**Findings:**

In this cross-sectional study of 10 191 individuals in Denmark with a first-time referral for diagnostic evaluation for dementia, those with a household income in the upper tertile were less likely to receive a dementia diagnosis after referral and had a less severe cognitive stage at diagnosis compared with individuals with a household income in the middle and lower tertiles.

**Meaning:**

Findings from this study suggest that, in Denmark, affluent individuals may have the advantage of receiving earlier dementia diagnosis.

## Teaser

This cross-sectional study describes the development of dementia and the level of cognitive decline among individuals with varying socioeconomic backgrounds who underwent a diagnostic evaluation for dementia.

## Introduction

Dementia is a medical condition characterized by cognitive functional decline that affects daily life and social activities.^1^ Approximately 50 million people worldwide live with dementia, and this number is expected to triple by 2050 with the aging of the population presenting a substantial challenge to patients, families, and society.^2^

Several risk factors for the development of dementia have been identified, including biological, lifestyle, environmental, and pathological factors associated with certain medical conditions and diseases.^3,4,5,6,7^ Socioeconomic status (SES), commonly measured by educational level, income level, and occupation, has been recognized as a risk factor for dementia and dementia-related death given that low SES was found to be associated with an increased risk for both.^8,9,10,11,12,13^

Aside from studies on the risk of developing dementia, few studies have focused on SES and its association with dementia diagnosis and cognitive severity at diagnosis. Findings of such studies generally showed that lower SES was associated with a higher risk of receiving a dementia diagnosis and lower cognitive function at the time of diagnosis.^14,15,16^ Although SES measures in these previous studies varied, the limitations in data collection and sample size in these studies as well as the discrepancies in health care access (eg, using free and universal or fee-based insurance) in Denmark compared to these countries may result in differences in association estimation. Moreover, most studies on SES have focused primarily on education and occupation as measures; however, household income (HHI) and wealth may be better indicators of SES for examining the health outcomes of older people who are retired or close to retirement, especially because education is typically pursued early in life.^12,17,18,19^ However, to our knowledge, no register-based nationwide study has been conducted on HHI and its association with the diagnostic evaluation for dementia.

Denmark offers universal, free health care services to all citizens regardless of their social or economic position.^20^ Danish national registries record health, social, and economic data and can be linked at the individual level.^21^ Herein, we conducted a nationwide study of individuals in Denmark who had a referral for a first-time diagnostic evaluation for dementia in 2017 to 2018. Our objective was to investigate whether HHI is associated with dementia diagnosis and cognitive severity at time of diagnosis.

## Methods

### Design, Data Sources, and Population

This population- and register-based cross-sectional study used data from Danish national registers. In compliance with European data protection rules, the University of Southern Denmark registered this project. According to Danish law, review by an ethics board and patient informed consent are not required for purely register-based studies. We followed the Strengthening the Reporting of Observational Studies in Epidemiology (STROBE) reporting guideline.^22^

Dementia-related health data, including diagnosis and cognitive stage at diagnosis, were retrieved from the Danish Quality Database for Dementia (DANDEM).^23^ The purpose of DANDEM is to improve and monitor the quality of diagnosis for all persons with a referral for elective dementia assessments to the Danish secondary health care sector, including memory clinics and dementia assessment units. DANDEM was established in January 2016, and the first year of data collection was considered as a trial period. Using the unique civil registration number assigned to Danish citizens at birth and to persons with a Danish residence permit on immigration, we linked individuals’ health data in DANDEM with HHI and household type from Statistics Denmark,^24^ demographic characteristics (age, sex, region of residency, and vital status, such as date of death) from the Danish Civil Registration System,^25^ educational level from the Population Education Register,^26^ history of medical conditions from the Danish National Patient Registry,^27^ and history of medications from the Danish National Prescription Registry.^28^

The study population was selected from 17 292 individuals with a referral to memory clinics or dementia assessment units across all regions of Denmark for a dementia diagnostic evaluation between January 1, 2017, and December 17, 2018. For this analysis, we included only individuals aged 45 years or older with a first-time referral for dementia evaluation and for whom complete data were registered. Complete data included dementia diagnosis, cognitive severity stage, and HHI during the study period (Figure). Data analysis was conducted from October 2019 to December 2020.

**Figure.**
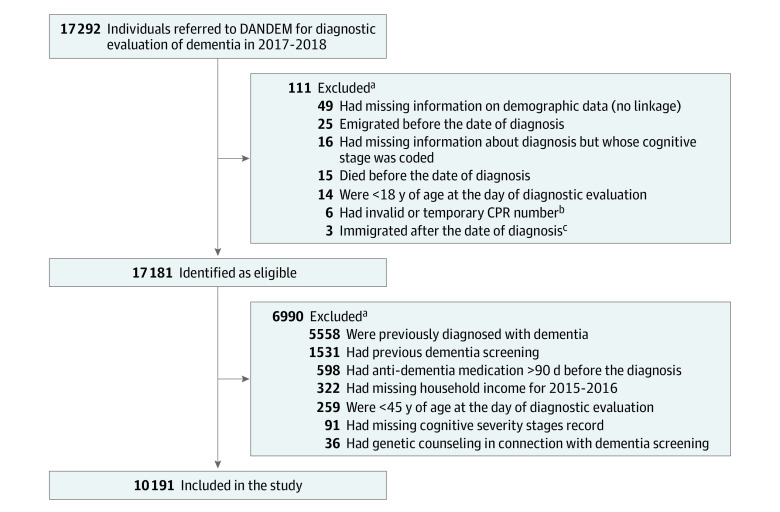
Flowchart of Population Selection ^a^Some individuals satisfied more than 1 exclusion criteria. ^b^Temporary civil registration number (CPR) refers to the identification number recorded in the Danish Quality Database for Dementia (DANDEM) that was a mix of letters and numbers. ^c^Data were missing before the date of diagnosis.

### Assessment of Household Income, Dementia Diagnosis, and Cognitive Stage at Diagnosis 

Annual HHI registered by Statistics Denmark is based on equivalized disposable income for the household.^29^ We used HHI as a proxy for SES. To avoid an illness factor in income, we retrieved HHI at 2 years before the dementia evaluation: 2015 HHI data were used for people with a referral for dementia evaluation in 2017, and 2016 HHI data were used for those with a referral in 2018. We categorized individuals according to income tertiles (lower, medium, or upper) within age groups. We used a different method to define personal income to investigate the referral rate (eMethods in the Supplement).

The date of dementia diagnosis, whether a dementia diagnosis was received (yes or no), type of dementia, and cognitive stage at diagnosis were retrieved from DANDEM. The latter 3 data points followed the *International Statistical Classification of Diseases and Related Health Problems, Tenth Revision* dementia diagnostic criteria.^30^ The types of dementia diagnosis in DANDEM are recorded as follows: Alzheimer disease, vascular dementia, mixed dementia (Alzheimer disease and vascular dementia), dementia with Lewy bodies, Parkinson disease dementia, or other types. Stages of cognitive severity in DANDEM are graded as follows: cognitively intact, mild cognitive impairment but not dementia, mild dementia, moderate dementia, or severe dementia. For the purposes of analysis, we extracted these stages and assigned a level to each stage as follows: level 1 was cognitively intact, level 2 was mild cognitive impairment but not dementia, level 3 was mild dementia, level 4 was moderate dementia, and level 5 was severe dementia.

### Assessment of Other Covariates

Based on previous findings,^31,32,33,34,35^ we included the baseline covariates of age, sex, household type, length of education (in years), region of residence, medication type, and medical conditions. We divided individuals into 6 age groups within 5-year intervals (<65, 65-69, 70-74, 75-79, 80-84, or ≥85 years). Household type was either living alone or living with someone. In accordance with the highest educational level recorded in the Population Education Register,^26^ we used the following categories for length of education: short term (≤10 years), medium term (11-15 years), or long term (>15 years).

Using *International Statistical Classification of Diseases and Related Health Problems, Tenth Revision* codes, we extracted from the Danish National Patient Registry each individual’s medical conditions for 10 years before the index date.^27^ Fourteen medical conditions were identified: type 2 diabetes, chronic obstructive pulmonary disease, ischemic heart disease, depression, hypertension, stroke, atrial fibrillation, cancer, fractures, peripheral vascular disease, hemorrhage, cerebrovascular disease, kidney disease, and rheumatic disease. These medical conditions have been found to be associated with dementia and household economics.^36,37,38,39,40,41^ Based on the presence of these medical conditions during the 10-year period, we grouped individuals according to the number of medical conditions they had (0, 1, 2, 3, or ≥4) in any combination.

We assessed 5 types of prescription medications registered in the Danish National Prescription Registry at 1 year before the index date^28^: antipsychotics, antianxiety, hypnotics and sedatives, antidepressants, and opioids. Because of the behavioral and psychological symptoms of dementia, these medicines are frequently prescribed to individuals with dementia.^42,43,44^

### Statistical Analysis

The frequency of the sample’s baseline characteristics as well as the dementia diagnosis and cognitive stage at diagnosis were described by HHI levels. We applied 4 logistic regression models to estimate the association between HHI (with the lower-tertile HHI being the reference group) and receipt of dementia diagnosis (yes or no). Model 1 was the crude analysis of HHI for dementia diagnosis. Model 2 started with model 1 and was adjusted for confounders (age group, sex, region of residence, household type, and year 2017 and 2018). Model 3 used model 2 as the basis and was adjusted for the 5 selected medication types. Model 4 was model 3 but adjusted for the 14 medical conditions and the number of medical conditions. Odds ratios (ORs) with 95% CIs were reported. We conducted supplementary analyses of the equality of estimation coefficients for HHI groups between model 1 and model 4, and we looked into the misclassification errors using model 4 as the sample data with and without HHI.

We applied linear regression models for estimating the association between HHI and cognitive severity stage at diagnosis in the crude (univariable) and multivariable models, adjusting for covariates in the 4 models. Coefficient (β) estimates with 95% CI were reported. All analyses were performed using Stata, version 16 (StataCorp LLC). A 2-tailed *P* < .05 was considered to be statistically significant.

## Results

Of 17 292 individuals with a first-time referral for diagnostic evaluation for dementia in 2017 to 2018, a total of 10 191 individuals (mean [SD] age, 75 [10] years; 5476 women [53.7%] and 4715 men [46.3%]) were eligible for analysis (Figure and Table 1). The number of individuals with HHI in the lower, middle, and upper tertiles was similar across age groups. However, families with higher SES had the lowest proportion of referrals for diagnostic evaluation for dementia (eTable 1 in the Supplement). Overall, individuals with HHI in the lower tertile vs the upper tertile were more likely to be women (2048 [60.3%] vs 1572 [46.3%]), to have a lower educational level (short term: 1716 [50.5%] vs 511 [15.1%]), to live alone (2093 [61.6%] vs 1121 [33.0%]), and to have multiple medical conditions (≥4: 717 [21.1%] vs 606 [17.9%]) (Table 1).

**Table 1.** Characteristics of Individuals With a First-Time Referral for Dementia Diagnostic Evaluation^a^

Variable	No. (%)			
	All (N = 10 191)	Household income^b^		
		Lower tertile (n = 3399)	Middle tertile (n = 3398)	Upper tertile (n = 3394)
Age, mean (SD), y	75 (10)	75 (10)	75 (10)	75 (10)
Age group, y				
<65	1523 (14.9)	508 (14.9)	508 (14.9)	507 (14.9)
65-69	987 (9.7)	329 (9.7)	329 (9.7)	329 (9.7)
70-74	1804 (17.7)	602 (17.7)	601 (17.7)	601 (17.7)
75-79	2075 (20.4)	692 (20.4)	692 (20.4)	691 (20.4)
80-84	2057 (20.2)	686 (20.2)	686 (20.2)	685 (20.2)
≥85	1745 (17.1)	582 (17.1)	582 (17.1)	581 (17.1)
Sex				
Male	4715 (46.3)	1351 (39.7)	1542 (45.4)	1822 (53.7)
Female	5476 (53.7)	2048 (60.3)	1856 (54.6)	1572 (46.3)
Period				
2017	4302 (42.2)	1534 (45.1)	1373 (40.4)	1395 (41.1)
2018	5889 (57.8)	1865 (54.9)	2025 (59.6)	1999 (58.9)
Length of education				
Short term (≤10 y)	3555 (34.9)	1716 (50.5)	1328 (39.1)	511 (15.1)
Medium term (11-15 y)	4685 (46.0)	1411 (41.5)	1641 (48.3)	1633 (48.1)
Long term (>15 y)	1718 (16.9)	166 (4.9)	361 (10.6)	1191 (35.1)
Unknown or missing data	233 (2.3)	106 (3.1)	68 (2.0)	59 (1.7)
Household type				
Living alone	4914 (47.2)	2093 (61.6)	1600 (47.1)	1121 (33.0)
Living with someone	5377 (52.8)	1306 (38.4)	1798 (52.9)	2273 (67.0)
Region of residence				
Region of Northern Denmark	648 (6.4)	268 (7.9)	203 (6.0)	177 (5.2)
Central Denmark Region	2025 (19.9)	647 (19.0)	716 (21.1)	662 (19.5)
Region of Southern Denmark	2990 (29.3)	1204 (35.4)	1042 (30.7)	744 (21.9)
Capital Region of Denmark	3328 (32.7)	904 (26.6)	1000 (29.4)	1424 (42.0)
Region Zealand	1200 (11.8)	376 (11.1)	437 (12.9)	387 (11.4)
Medical conditions				
COPD	1106 (10.9)	433 (12.7)	401 (11.8)	272 (8.0)
Type 2 diabetes	1300 (12.8)	513 (15.1)	439 (12.9)	348 (10.3)
Cancer	1168 (11.5)	352 (10.4)	400 (11.8)	416 (12.3)
Hypertension	3650 (35.8)	1328 (39.1)	1232 (36.3)	1090 (32.1)
Depression	1172 (11.5)	438 (12.9)	408 (12.0)	326 (9.6)
Fractures	5215 (51.2)	1699 (50.0)	1752 (51.6)	1764 (52.0)
Stroke	961 (9.4)	324 (9.5)	325 (9.6)	312 (9.2)
Ischemic heart condition	1511 (14.8)	531 (15.6)	524 (15.4)	456 (13.4)
Atrial fibrillation	1447 (14.2)	493 (14.5)	483 (14.2)	471 (13.9)
Peripheral vascular disease	479 (4.7)	180 (5.3)	156 (4.6)	143 (4.2)
Hemorrhage	1102 (10.8)	379 (11.2)	358 (10.5)	365 (10.8)
Cerebrovascular disease	1748 (17.2)	590 (17.4)	594 (17.5)	564 (16.6)
Kidney disease	342 (3.4)	137 (4.0)	109 (3.2)	96 (2.8)
Rheumatic disease	369 (3.6)	121 (3.6)	127 (3.7)	121 (3.6)
No. of medical conditions				
0	1727 (16.9)	545 (16.0)	558 (16.4)	624 (18.4)
1	2722 (26.7)	841 (24.7)	891 (26.2)	990 (29.2)
2	2116 (20.8)	745 (21.9)	694 (20.4)	677 (19.9)
3	1528 (15.0)	533 (15.7)	517 (15.2)	478 (14.1)
≥4	2036 (20.0)	717 (21.1)	713 (21.0)	606 (17.9)
Unknown or missing data	62 (0.6)	18 (0.5)	25 (0.7)	19 (0.6)
Medication type				
Antipsychotics	602 (5.9)	244 (7.2)	203 (6.0)	155 (4.6)
Antianxiety	502 (4.9)	200 (5.9)	177 (5.2)	125 (3.7)
Hypnotics and sedatives	972 (9.5)	315 (9.3)	331 (9.7)	326 (9.6)
Antidepressants	2489 (24.4)	895 (26.3)	878 (25.8)	716 (21.1)
Opioids	1439 (14.1)	536 (15.8)	513 (15.1)	390 (11.5)

Abbreviation: COPD, chronic obstructive pulmonary disease.

^a^ From January 1, 2017, to December 17, 2018.

^b^ Household income in tertiles within age groups.

Table 2 shows that, among the 8844 of 10 191 individuals (86.8%) who received a dementia diagnosis, fewer had an HHI in the upper tertile (2839 [83.6%]) than in the middle (2989 [88.0%]) and lower (3016 [88.7%]) tertiles. Also, fewer individuals with upper-tertile HHI presented with moderate (748 [22.0%]) or severe (147 [4.3%]) dementia at the time of diagnosis compared with individuals with middle-tertile (moderate dementia: 882 [26.0%]; severe dementia: 185 [5.4%]) and lower-tertile (moderate dementia: 902 [26.5%]; severe dementia: 193 [5.7%]) HHI.

**Table 2.** Distribution of Individuals by Dementia Diagnosis, Dementia Type, and Cognitive Severity Stage at Diagnosis^a^

Variable	No. (%)			
	All (N = 10 191)	Household income^b^		
		Lower tertile (n = 3399)	Middle tertile (n = 3398)	Upper tertile (n = 3394)
Received a dementia diagnosis?				
No	1347 (13.2)	383 (11.3)	409 (12.0)	555 (16.4)
Yes	8844 (86.8)	3016 (88.7)	2989 (88.0)	2839 (83.6)
Dementia type				
Alzheimer disease	3204 (31.4)	1042 (30.7)	1120 (33.0)	1042 (30.7)
Vascular dementia	908 (10.3)	300 (9.9)	332 (11.1)	276 (9.7)
Mixed dementia^c^	765 (7.5)	275 (8.1)	267 (7.9)	223 (6.6)
Dementia with Lewy bodies	280 (2.7)	82 (2.4)	82 (2.4)	116 (3.4)
PD dementia	127 (1.2)	34 (1.0)	52 (1.5)	41 (1.2)
Frontotemporal dementia	179 (1.8)	49 (1.4)	52 (1.5)	78 (2.3)
Normal pressure hydrocephalus	175 (1.7)	50 (1.5)	66 (1.9)	59 (1.7)
Huntington disease	24 (0.2)	7 (0.2)	6 (0.2)	11 (0.3)
Other neurodegenerative disease	154 (1.5)	43 (1.3)	41 (1.2)	70 (2.1)
Unresolved cause	1555 (15.3)	520 (15.3)	514 (15.1)	521 (15.4
Alcohol-related dementia	258 (2.5)	134 (3.9)	72 (2.1)	52 (1.5)
Other not neurodegenerative disease	712 (7.0)	273 (8.0)	219 (6.4)	220 (6.5)
Affective disorder	503 (4.9)	207 (6.1)	166 (4.9)	130 (3.8)
Cognitive severity stage at diagnosis				
Cognitively intact	1430 (14.0)	407 (12.0)	423 (12.4)	600 (17.7)
MCI but not dementia	2943 (28.9)	1028 (30.2)	937 (27.6)	978 (28.8)
Mild dementia	2761 (27.1)	869 (25.6)	971 (28.6)	921 (27.1)
Moderate dementia	2532 (24.8)	902 (26.5)	882 (26.0)	748 (22.0)
Severe dementia	525 (5.2)	193 (5.7)	185 (5.4)	147 (4.3)

Abbreviations: MCI, mild cognitive impairment; PD, Parkinson disease.

^a^ From January 1, 2017, to December 17, 2018.

^b^ Household income in tertiles within age groups.

^c^ Alzheimer disease and vascular dementia.

Logistic regression analyses showed that, compared with individuals with lower-tertile HHI, the odds ratio (OR) for receiving a dementia diagnosis was 0.90 (95% CI, 0.77-1.05) for individuals with HHI in the middle tertile and was 0.66 (95% CI, 0.57-0.77) for those in the upper tertile (Table 3). After successive adjustment for the covariates (model 4), the ORs were attenuated but remained similar, indicating a significantly lower risk for those with upper-tertile HHI (OR, 0.65; 95% CI, 0.55-0.78) and not significantly higher risk for individuals with middle-tertile HHI (OR, 0.92; 95% CI, 0.77-1.09). Furthermore, supplementary analyses showed no statistical difference between the estimated coefficients for HHI groups between model 1 and model 4. Misclassification error analysis using model 4 as the sample data indicated that HHI was a relevant factor for dementia diagnosis in this study population but was not a main factor (eTable 2 and eFigure in the Supplement).

**Table 3.** Association Between Household Income and Dementia Diagnosis and Cognitive Severity Stage at Diagnosis^a^

Household income	Model 1^b^	Model 2^c^	Model 3^d^	Model 4^e^
Logistic regressions for dementia diagnosis, OR (95% CI)				
Lower tertile	1 [Reference]	1 [Reference]	1 [Reference]	1 [Reference]
Middle tertile	0.90 (0.77 to 1.05)	0.92 (0.78 to 1.09)	0.92 (0.78 to 1.09)	0.92 (0.77 to 1.09)
Upper tertile	0.66 (0.57 to 0.77)	0.67 (0.56 to 0.79)	0.67 (0.57 to 0.80)	0.65 (0.55 to 0.78)
Linear regressions for cognitive severity stage at diagnosis, β (95% CI)				
Lower tertile	1 [Reference]	1 [Reference]	1 [Reference]	1 [Reference]
Middle tertile	−0.00 (−0.06 to 0.05)	0.00 (−0.05 to 0.05)	0.01 (−0.04 to 0.06)	0.01 (−0.04 to 0.06)
Upper tertile	−0.16 (−0.21 to −0.10)	−0.15 (−0.20 to −0.10)	−0.15 (−0.20 to −0.10)	−0.16 (−0.21 to −0.10)
Constant	1.85 (1.81 to 1.89)	−1.40 (−1.88 to −0.92)	−1.42 (−1.90 to −0.94)	−1.29 (−1.77 to 0.81)

Abbreviation: OR, odds ratio.

^a^ Among 9203 individuals; see model 4 description for the complete information on any covariates.

^b^ Model 1 was the crude model of household income for dementia diagnosis.

^c^ Model 2 was model 1 adjusted for age group, sex, region of residence, household type, and period (2017 and 2018).

^d^ Model 3 was model 2 adjusted for 5 types of medications (antipsychotics, antianxiety, hypnotics and sedatives, antidepressants, and opioids).

^e^ Model 4 was model 3 adjusted for 14 medical conditions (type 2 diabetes, chronic obstructive pulmonary disease, ischemic heart disease, depression, hypertension, stroke, atrial fibrillation, cancer, fractures, peripheral vascular disease, hemorrhage, cerebrovascular disease, kidney disease, and rheumatic disease) and number of medical conditions (0, 1, 2, 3, or ≥4) in any combination.

When compared with lower-tertile HHI, middle-tertile HHI was not associated with cognitive severity at diagnosis (β, 0.01; 95% CI, −0.04 to 0.06); however, upper-tertile HHI was significantly inversely associated with cognitive severity stage at diagnosis (β, −0.16; 95% CI, −0.21 to −0.10) (model 4 in Table 3). These associations remained similar when counting only the individuals who were diagnosed with mild, moderate, or severe dementia and excluding individuals with other cognitive stages that were present at time of diagnosis (eTable 3 in the Supplement).

## Discussion

Using HHI as a proxy for SES, we found that individuals with higher SES were less likely to be diagnosed with dementia and, if diagnosed, had less severe dementia stage. Individuals with mid-level SES did not differ from individuals with lower SES in terms of dementia diagnosis and cognitive severity stage at diagnosis.

Previous studies frequently reported that lower SES was associated with a higher risk of dementia diagnosis and a later stage of dementia at diagnosis and that individuals with higher SES were less likely to be diagnosed with dementia but, if diagnosed, had less severe dementia.^14,15,16,45,46^ A small Canadian study (262 patients from a memory clinic) reported that individuals with lower SES presented at the clinic later and with more advanced dementia compared with those with higher SES.^14^ In a larger study from the US (1658 individuals with Alzheimer disease), having fewer years of education was associated with later detection of the disease and a more severe stage at diagnosis.^15^ A study from England (1420 patients with new referrals) showed that socioeconomic deprivation (based on residential postal codes) was associated with lower cognitive function compared with living in more affluent areas.^16^

Discrepancies in study findings may be a natural consequence of the different measurement protocols for SES. The aforementioned studies often used educational level as a measure for SES. In a Swedish study, Darin-Mattsson and colleagues^19^ reported that income was associated with late-life health that was independent of all other SES indicators, including educational level, occupational complexity, social class, and SES index (the summary of all 5 indicators). In the present study, most of the individuals were pensioners, and the tax-registered pension was the primary income. We used HHI as a measurement of SES and found that, although lower HHI was a risk factor for dementia diagnosis and severity stage at diagnosis, people with higher HHI did benefit from health inequality; they received earlier referrals and earlier diagnosis, which again benefitted them as they were able to get earlier treatment and potentially slow the disease progression compared with individuals with lower SES. Adding education into this analysis did not change these findings (eTable 4 in the Supplement). Although education has been associated with cognitive function and has been suggested to supply cognitive reserve,^47^ judging from current empirical evidence, its contribution to cognitive reserve or decline is limited.^48,49^

People with higher SES often have higher educational attainment and higher paying jobs that require certain levels of intellectual function; perhaps individuals with more cognitively demanding jobs can more easily perceive their own cognitive changes, thus leading them to consult with a physician earlier. In addition, higher educational level not only has been associated with a reduced risk of dementia but is believed to lead to better understanding of dementia and better awareness of the symptoms for early detection and diagnosis.^14,50^ Systematic reviews have found that educational deficits were a major factor in dementia diagnosis delay^51^ and that other factors, such as fear of treatment result, denial, dementia stigma, and living alone, can also delay individuals from seeking a diagnostic evaluation for dementia.^52,53,54^ Moreover, lack of awareness and/or lack of knowledge of dementia may also be factors in greater severity at the time of diagnosis for people with lower SES.^55,56^

In addition, we conducted another analysis that showed that families with higher SES had the lowest proportion of referrals for diagnostic evaluation for dementia (eTable 1 in the Supplement). Although referrals can be changed by health care systems,^57^ referrals are commonly the product of the assessment by a general practitioner combined with disease symptoms, patient preference, and patient ability to share information and derive maximum value from communication, examination, and experiences.^58^ It has been argued that studies based on patient referrals to secondary or tertiary care centers may have severe selection bias.^59^

Although we conducted a population-based study using dementia diagnoses from all memory clinics and dementia assessment units located in all regions of Denmark, it is unknown how many individuals were diagnosed with dementia by their general practitioner without need for a further referral. Many health care professionals hypothesized that dementia would be undetectable and underdiagnosed in many countries, including Denmark,^60,61^ and that a higher proportion of such undetected cases would likely consist of individuals with lower SES.^62^ However, this hypothesis needs to be investigated further.

### Strengths and Limitations

This study has some strengths. To our knowledge, this analysis is the first population- and register-based study of HHI as a proxy for SES and its association with diagnostic evaluation for dementia. By using different degrees of analysis models, we found an association between HHI and dementia diagnosis and cognitive severity stage at diagnosis in the context of a universal health care system.

This study also has some limitations. The health data related to referrals, dementia diagnosis, and cognitive severity stage were obtained from DANDEM, a high-quality national database for dementia established in 2016 to improve and monitor the quality of diagnosis at memory clinics or dementia assessment units throughout Denmark. A 2015 study of the memory clinics in the Capital Region of Denmark reported that limited resources in memory clinics may have been a factor in the impaired precision of dementia diagnosis.^63^ To our knowledge, no study has yet performed a comprehensive evaluation or validation of DANDEM for the diagnoses recorded by these memory clinics and assessment units; further research is, therefore, warranted.

We did not adjust for lifestyle factors, such as smoking, diet, alcohol consumption, and physical activity, because Denmark generally does not register such information in the national registers; however, these factors are associated with SES and are often considered to be modifiable factors in the risk of dementia.^64,65^ Adding these factors into analysis when they become available may strengthen the accuracy of association estimation.

The data set we used included only cross-sectional data on individuals who received a first-time referral for a diagnostic evaluation for dementia in the secondary health care sector in 2017 to 2018 and registered in DANDEM. Furthermore, we had no information on onset of symptoms, date of physician visit, or date of referral. Therefore, the data did not allow longitudinal follow-up, including the 4-stage timeline of disease progression from neuropathology with no clinical signs to late-stage disease.^66^ A 2014 survey-based report of participant responses from 24 European countries revealed that 81% of the respondents spent an average of at least 8 weeks from time of referral to specialist visit for assessment; the other respondents reported 4 weeks or less.^52^ However, it may take up to 2 years for family members to notice the first symptoms of dementia before the consultation and about 3 years before diagnosis.^67^ We were unclear about the implication of SES for the 4 stages of dementia progression in the Danish context. Further studies should take this impact into consideration.

## Conclusions

In this population- and register-based cross-sectional study of individuals with referrals for a dementia diagnostic evaluation in Denmark, SES was associated with dementia diagnosis and cognitive severity at diagnosis, especially for those in higher income households. Individuals with higher SES were less likely to receive a dementia diagnosis and, among those diagnosed with dementia, had less severe cognitive stage at diagnosis compared with individuals with lower SES. These results reveal a social inequity in diagnostic evaluation for dementia in Denmark: affluent individuals seem to have the advantage of receiving earlier diagnosis. Public health strategies should target people with lower SES for earlier detection and intervention for dementia.

## Notes

Supplement. **eMethods.** Statistical Methods for Defining Individual Personal Income Using Household Income **eTable 1.** Referral Rates by Adjustable Household Income Group **eTable 2.** Estimated Mean Sensitivity, Specificity, Positive Predictive Value, Negative Predictive Value, and Misclassification Error Based on 10-Fold Cross-Validation for Logistic Model 4 With and Without Household Income (HHI) **eTable 3.** Linear Regression of the Association Between Household Income and Cognitive Severity Stage at Diagnosis **eTable 4.** Association Between Household Income and Dementia Diagnosis and Severity at Diagnosis in the Full Adjusted Model **eFigure.** ROC Curve for Misclassification Error Using Model 4 Dataset With and Without Household Income
