# Supplementary material for: Association of Socioeconomic Status With Dementia Diagnosis Among Older Adults in Denmark
Source: JAMA Netw Open. 2021 May 18;4(5):e2110432. doi: 10.1001/jamanetworkopen.2021.10432 (PMC8132141; doi:10.1001/jamanetworkopen.2021.10432)
Supplement: Supplement. — eMethods. Statistical Methods for Defining Individual Personal Income Using Household Income eTable 1. Referral Rates by Adjustable Household Income Group eTable 2. Estimated Mean Sensitivity, Specificity, Positive Predictive Value, Negative Predictive Value, and Misclassification Error Based on 10-Fold Cross-Validation for Logistic Model 4 With and Without Household Income (HHI) eTable 3. Linear Regression of the Association Between Household Income and Cognitive Severity Stage at Diagnosis eTable 4. Association Between Household Income and Dementia Diagnosis and Severity at Diagnosis in the Full Adjusted Model eFigure. ROC Curve for Misclassification Error Using Model 4 Dataset With and Without Household Income [file jamanetwopen-e2110432-s001.pdf]

Supplementary Online Content

Petersen JD, Wehberg S, Packness A, et al. Association of socioeconomic status with dementia diagnosis among older adults in Denmark. *JAMA Netw Open*. 2021;4(5):e2110432. doi:10.1001/jamanetworkopen.2021.10432

eMethods. Statistical Methods for Defining Individual Personal Income Using Household Income

eTable 1. Referral Rates by Adjustable Household Income Group

eTable 2. Estimated Mean Sensitivity, Specificity, Positive Predictive Value, Negative Predictive Value, and Misclassification Error Based on 10-Fold Cross-Validation for Logistic Model 4 With and Without Household Income (HHI)

eTable 3. Linear Regression of the Association Between Household Income and Cognitive Severity Stage at Diagnosis

eTable 4. Association Between Household Income and Dementia Diagnosis and Severity at Diagnosis in the Full Adjusted Model

eFigure. ROC Curve for Misclassification Error Using Model 4 Dataset With and Without Household Income

This supplementary material has been provided by the authors to give readers additional information about their work.

© 2021 Petersen JD et al. *JAMA Network Open.*

eMethods. Statistical Methods for Defining Individual Personal Income Using Household Income

We wanted to illustrate the effect of income category on the referral rate for dementia screening but had no access to data on an individual level to define the denominator. To come as close as possible with the data available (that is, similar to aggregated data available at https://www.statistikbanken.dk/), we employed the following strategy:

For the denominator, the number of families by adjusted disposable HHI (based on the variable famdisponibel_13) was extracted from the table INDKF132 for year 2017. HHI was grouped into "<200.000 kr.", "200.000-300.000 kr." and ">=300.000 kr." (kr.: Danish crowns). For the nominator, we assumed each referral was from a separate household. We converted the HHI available in our population (variable famaekvivadisp_13) to approximate the adjusted disposable HHI using the formula: adjusted disposable HHI = famakvivadisp_13 * (1 + 0.5 * x), with x being zero if the person was categorized as living alone, and one if the person was categorized as living with someone. The approximation assigns individuals living alone and individuals living with one other person (above the age of 14) the correct adjusted disposable HHI; the approximation is too low, however, for individuals living with multiple persons above the age of 14.

© 2021 Petersen JD et al. *JAMA Network Open.*

eTable 1. Referral Rates by Adjustable Household Income Group

P-value of Chi-squared test <.001.

**Adjustable household income group**	**Number of families**	**Referred to dementia screening**	**Referral per 1000 families**
<200.000 kr. (Danish crowns)	927453	3404	3,67
200.000-300.000 kr.	646152	3254	5,04
>300.000 kr.	1399426	3533	2,52
**Total**	2973031	10191	3,43

© 2021 Petersen JD et al. *JAMA Network Open.*

eTable 2. Estimated Mean Sensitivity, Specificity, Positive Predictive Value, Negative Predictive Value, and Misclassification Error Based on 10-Fold Cross-Validation for Logistic Model 4 With and Without Household Income (HHI)

The classification cutpoint for the logistic prediction was chosen as success probability in the respective Model 4 training data (range 0.86-0.88). Sample size were 920 or 921.

Model	Sensitivity	Specificity	False positive	False negative	Misclassification Error
M4-with HHI	0.713	0.686	0.314	0.287	0.291
M4-without HHI	0.712	0.681	0.319	0.288	0.292

© 2021 Petersen JD et al. *JAMA Network Open.*

eTable 3. Linear Regression of the Association Between Household Income and Cognitive Severity Stage at Diagnosis

Household income	Model 1	Model 2	Model 3	Model 4
	β (95% CI)			
**Cognitive severity stage excluding cognitively intact (n=7984)**				
Lower tertile	Reference	Reference	Reference	Reference
Middle tertile	0.02 (-0.03 to 0.07)	0.01 (-0.04 to 0.06)	0.01 (-0.03 to 0.06)	0.01 (-0.03 to 0.06)
Upper tertile	-0.06 (-0.11 to -0.01)	-0.09 (-0.14 to -0.04)	-0.09 (-0.14 to -0.04)	-0.09 (-0.14 to -0.04)
Cons	2.08 (2.05 to 2.12)	-0.53 (-1.03 to -0.03)	-0.53 (-1.02 to -0.03)	-0.43 (-0.93 to 0.06)
**Cognitive severity stages excluding cognitively intact or MCI (n=5292)**				
Lower tertile	0 (Reference)	0 (Reference)	0 (Reference)	0 (Reference)
Middle tertile	-0.03 (-0.07 to 0.01)	-0.03 (-0.07 to 0.01)	-0.03 (-0.07 to 0.01)	-0.03 (-0.08 to 0.01)
Upper-tertile	-0.07 (-0.11 to -0.03)	-0.08 (-0.13 to -0.04)	-0.08 (-0.13 to -0.04)	-0.09 (-0.13 to -0.04)
Cons	2.65 (2.62 to 2.68)	1.16 (0.65 to 1.66)	1.10 (0.60 to 1.59)	1.09 (0.59 to 1.59)

Model 1 was the crude model for household income for dementia diagnosis.
Model 2 was model 1 adjusted for age group, sex, region of residence, household type, and period (2017 and 2018).
Model 3 was model 2 adjusted for five types of medications (antipsychotics, antianxiety, hypnotics and sedatives, antidepressants, and opioids).
Model 4 was model 3 adjusted for 14 medical conditions (type 2 diabetes, chronic obstructive pulmonary disease, ischemic heart disease, depression, hypertension, stroke, atrial fibrillation, cancer, fractures, peripheral vascular disease, hemorrhage, cerebrovascular disease, kidney disease, and rheumatic disease) and number of medical conditions (0, 1, 2, 3, or ≥4) in any combination.

© 2021 Petersen JD et al. *JAMA Network Open.*

eTable 4. Association Between Household Income and Dementia Diagnosis and Severity at Diagnosis in the Full Adjusted Model[a] (n=9203[b])

Variable	Logistic regression between HHI and dementia diagnosis	Linear regression between HHI and severity at diagnosis
	OR (95% CI)	β (95% CI)
**Household income (HHI)**		
Lower tertile	Ref	Ref
Middle tertile	0.95 (0.80 to 1.13)	0.02 (-0.03 to 0.07)
Upper tertile	0.74 (0.62 to 0.90)	-0.09 (-0.15 to -0.03)
**Age group,** y		
<65	Ref	Ref
65-69	1.22 (0.90 to 1.67)	0.07 (-0.05 to 0.19)
70-74	1.62 (1.11 to 2.37)	0.12 (-0.02 to 0.26)
75-79	2.17 (1.34 to 3.51)	0.21 (0.03 to 0.39)
80-84	2.77 (1.54 to 4.99)	0.26 (0.05 to 0.48)
≥85	3.58 (1.72 to 7.47)	0.30 (0.03 to 0.56)
**Sex**		
Male	Ref	Ref
Female	0.80 (0.70 to 0.92)	-0.03 (-0.08 to 0.01)
**Period**		
2017	Ref	Ref
2018	1.10 (0.96 to 1.25)	0.06 (0.01 to 0.10)
**Household type**		
Living alone	Ref	Ref
Living with someone	0.81 (0.70 to 0.95)	-0.11 (-0.15 to -0.06)
**Region of residence**		
Region of Northern Denmark	Ref	
Central Denmark Region	1.44 (1.07 to 1.93)	0.49 (0.40 to 0.58)
Region of Southern Denmark	1.33 (1.00 to 1.76)	0.37 (0.28 to 0.46)
Capital Region of Denmark	0.88 (0.67 to 1.15)	0.34 (0.25 to 0.43)
Region Zealand	1.18 (0.86 to 1.62)	0.37 (0.27 to 0.47)
**Medical conditions**		
Chronic obstructive pulmonary disease	0.82 (0.65 to 1.05)	-0.13 (-0.20 to -0.05)
Type 2 diabetes	1.26 (0.99 to 1.61)	0.04 (-0.03 to 0.11)
Cancer	1.30 (1.00 to 1.69)	-0.02 (-0.10 to 0.05)
Hypertension	1.25 (1.00 to 1.56)	-0.01 (-0.07 to 0.06)
Depression	0.73 (0.57 to 0.93)	-0.32 (-0.40 to -0.25)
Fractures	1.29 (1.05 to 1.59)	0.02 (-0.04 to 0.08)
Stork	1.65 (1.17 to 2.34)	0.24 (0.14 to 0.33)
Ischemic heart condition	0.91 (0.72 to 1.14)	-0.06 (-0.12 to 0.01)
Atrial fibrillation	1.42 (1.10 to 1.83)	0.08 (0.01 to 0.14)
Peripheral vascular disease	1.22 (0.86 to 1.71)	0.00 (-0.09 to 0.10)
Hemorrhage	1.44 (1.11 to 1.87)	0.13 (0.06 to 0.20)
Cerebrovascular disease	1.15 (0.86 to 1.54)	-0.05 (-0.13 to 0.04)
Kidney disease	0.94 (0.63 to 1.39)	0.01 (-0.10 to 0.12)
Rheumatic disease	0.94 (0.66 to 1.36)	-0.08 (-0.19 to 0.04)
**Number of medical conditions**		
0	Ref	
1	0.70 (0.54 to 0.92)	-0.09 (-0.17 to -0.01)
2	0.65 (0.45 to 0.95)	-0.11 (-0.22 to -0.00)
3	0.54 (0.32 to 0.89)	-0.13 (-0.27 to 0.02)
≥4	0.34 (0.16 to 0.70)	-0.16 (-0.36 to 0.05)

© 2021 Petersen JD et al. *JAMA Network Open.*

Variable	Logistic regression between HHI and dementia diagnosis	Linear regression between HHI and severity at diagnosis
	OR (95% CI)	β (95% CI)
**Medication**		
Antipsychotics N05A	1.45 (1.09 to 1.93)	0.21 (0.12 to 0.29)
Antianxiety N05B	1.37 (1.16 to 1.62)	0.07 (0.02 to 0.12)
Hypnotics and sedatives N05C	0.98 (0.74 to 1.31)	-0.09 (-0.19 to -0.00)
Antidepressants N06A	0.77 (0.62 to 0.95)	-0.20 (-0.27 to -0.13)
Opioids N02A	0.94 (0.78 to 1.14)	0.00 (-0.05 to 0.06)
**Education**		
Short (≤10 years)	Ref	Ref
Medium (11-15 years)	0.86 (0.73 to 1.02)	-0.07 (-0.12 to -0.03)
Long (>15 years)	0.64 (0.52 to 0.80)	-0.22 (-0.29 to -0.15)

[a]Adjusted with all covariates listed in this table.

[b]With complete information on any covariates listed in this table.

© 2021 Petersen JD et al. *JAMA Network Open.*

eFigure. ROC Curve for Misclassification Error Using Model 4 Dataset With and Without Household Income

Based on a random sample of 10% of the whole dataset

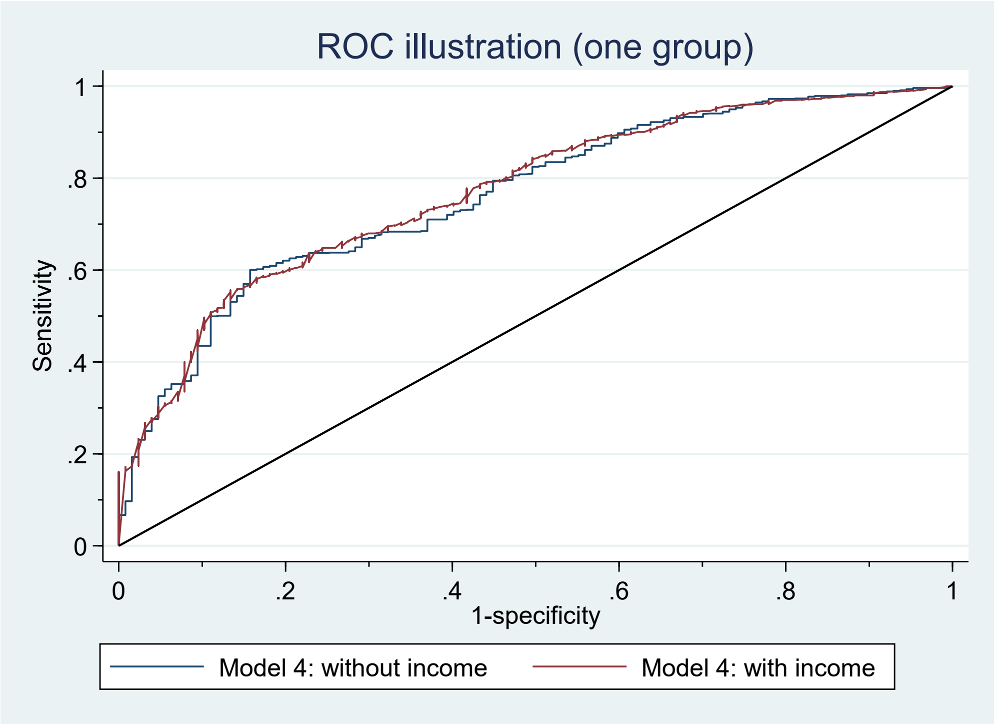

ROC: Receiver Operating Characteristics; Income: household income

By using one group of Model 4 dataset (10% of total Model 4 dataset), eFigure shows the two areas (the blue line represents Model 4 without HHI, and the red line represents Model 4 with HHI) under the ROC curves. The cures are quite similar, with a very small advantage for the red cure (estimated mean AUC across the 10-cross-validation dataset was 0.76 for the model without HHI and 0.77 for the model with HHI), which indicating that the HHI was a relevant factor but not a main driver for dementia diagnosis for this study population.

© 2021 Petersen JD et al. *JAMA Network Open.*
